# Photoluminescence Redistribution of InGaN Nanowires Induced by Plasmonic Silver Nanoparticles

**DOI:** 10.3390/nano13061069

**Published:** 2023-03-16

**Authors:** Talgat Shugabaev, Vladislav O. Gridchin, Sergey D. Komarov, Demid A. Kirilenko, Natalia V. Kryzhanovskaya, Konstantin P. Kotlyar, Rodion R. Reznik, Yelizaveta I. Girshova, Valentin V. Nikolaev, Michael A. Kaliteevski, George E. Cirlin

**Affiliations:** 1 Faculty of Physics, St. Petersburg State University, Universitetskaya Embankment 13B, 199034 St. Petersburg, Russia; talgashugabaev@gmail.com (T.S.); gridchinvo@gmail.com (V.O.G.); moment92@mail.ru (R.R.R.); 2 Department of Physics, Alferov University, Khlopina 8/3, 194021 St. Petersburg, Russia; 3 Institute for Analytical Instrumentation RAS, Rizhsky 26, 190103 St. Petersburg, Russia; 4 Department of Physics, Higher School of Economics, Kantemirovskaya 3/1 A, 194100 St. Petersburg, Russia; 5 Ioffe Institute, Polytechnicheskaya 26, 194021 St. Petersburg, Russia; demid.kirilenko@mail.ioffe.ru; 6 Department of Physics, ITMO University, Kronverkskiy pr. 49, 197101 St. Petersburg, Russia

**Keywords:** InGaN nanowires, molecular beam epitaxy, silver nanoparticles, hybrid nanostructures, Fröhlich resonance

## Abstract

Hybrid nanostructures based on InGaN nanowires with decorated plasmonic silver nanoparticles are investigated in the present study. It is shown that plasmonic nanoparticles induce the redistribution of room temperature photoluminescence between short-wavelength and long-wavelength peaks of InGaN nanowires. It is defined that short-wavelength maxima decreased by 20%, whereas the long-wavelength maxima increased by 19%. We attribute this phenomenon to the energy transfer and enhancement between the coalesced part of the NWs with 10–13% In content and the tips above with an In content of about 20–23%. A proposed Fröhlich resonance model for silver NPs surrounded by a medium with refractive index of 2.45 and spread 0.1 explains the enhancement effect, whereas the decreasing of the short-wavelength peak is associated with the diffusion of charge carriers between the coalesced part of the NWs and the tips above.

## 1. Introduction

InGaN nanowires (NWs) are promising solids for creating new generation light emitting diodes, e.g., [1,2,3,4]. Free lateral surfaces of NWs, a small footprint between NWs and an underlying substrate provide efficient strain relaxation on the NW sidewalls without forming structural defects. It was shown [5,6] that this process contributes to growing InGaN NWs over the entire compositional range, which allows one to fabricate monolithic RGB micro LEDs on a single substrate [5,7,8,9]. As a result, InGaN NWs can be grown on lattice-mismatched substrates, in particular on Si [7,10,11].

Metal nanoparticles (NPs) attract much attention because it is possible to control their plasmonic properties by changing the shape, size, and environment of NPs [12]. Nowadays, noble metal NPs are extensively used in sensorics [13], biomedicine [14], and theranostics [15]. Therefore, there are many ways to synthesize metal NPs: nanolithographic methods (electron beam, ion beam lithography, nanospheric lithography), gas-phase synthesis [16], electrochemical methods [17], laser ablation [18], colloid chemistry method [16], etc. The latter method is, in fact, one of the most popular among researchers due to its availability, relative simplicity, cheapness, and high quality of synthesized NPs. In addition, localized plasmon resonance (LPR) in metal NPs in combination with semiconductor materials can significantly increase the photoexcitation of the latter. Decorating the surface of a semiconductor with metal NPs promotes the injection of hot electrons from the metal [19,20] into the NWs and ensures plasmon-induced energy transfer [20,21] upon excitation of LPR in NPs. This makes metal NPs an important object for solving applied problems in the field of modern optoelectronics and nanophotonics. In particular, silver nanostructures allow one to enhance the luminescence properties of InGaN/GaN layers [22,23,24].

NW/NP hybrid nanostructures can be fabricated by combining the unique physical properties of semiconductor NWs with the exceptional properties of metal NPs. Such structures have a large degree of freedom in varying shapes, sizes, materials, geometry, etc. [25]. In particular, the deposition of Ag NPs on Si NWs results in a significant increase of photocurrent in comparison with the grown sample, as was shown in [26,27]. In addition to the increase in photoexcitation in semiconductor NWs, there are examples of luminescence enhancement; a sixfold increase in the photoluminescence (PL) of ZnO NWs was demonstrated using metal NPs [28]. Casadei A. et al. [29] showed the twenty-fold electric field enhancement inside the GaAs NW-Au nanoantennas gap regions, which is an important step for creating effective nonlinear optical devices, new solar cells and other applications. However, the methods of fabrication, architecture control and physical properties of hybrid nanostructures based on InGaN NWs decorated with Ag NPs have not been previously investigated.

In this work, we fabricate NW/NP hybrid nanostructures based on InGaN NWs and study their photoluminescent properties. The initial InGaN NWs contain two areas with unequal indium content, which appear in the PL spectrum as short-wavelength and long-wavelength maxima. We show that the deposition of Ag NPs on InGaN NWs results in the room temperature photoluminescence (RT PL) redistribution from the areas with 10–13% In content to the areas with 20–23% In content. A model was developed that explained this effect. The results open ways to increase the photoluminescence efficiency of the higher In content areas in InGaN NWs through plasmonic nanoparticles.

## 2. Materials and Methods

The InGaN NWs were grown on p-type Si(111) substrate using Riber Compact 12 MBE system equipped with In and Ga effusion cells and a nitrogen plasma source. Prior to the growth, the substrate was thermally treated at 950 °C to remove silicon oxide from the growth surface. The substrate temperature was then decreased to 655 °C. At this moment, an atomically clean growth surface was detected through in situ reflection high-energy electron diffraction showing (7 × 7) surface reconstruction. After stabilization of the substrate temperature, the nitrogen plasma source was ignited and the Ga and In shutters were simultaneously opened. The growth lasted 3 h. The nitrogen flux and power of the nitrogen plasma source were set at 0.4 sccm and 450 W, respectively. Beam equivalent pressures of In and Ga measured by the Bayard–Alpert vacuum gauge were equal to each other and amounted to 1 × 10^−7^ Torr. The growth was carried out under nitrogen-rich growth conditions to ensure 3D growth.

The first part of silver NPs was synthesized using the colloidal method in an aqueous medium based on [30,31]. CI^−^ ions were used to form spherical NPs. A typical synthesis process was as follows. The synthesis temperature was 90 °C. Distilled water was heated in a flask with active mixing at 700 rpm. Then, 50 µL of NaCl solution (85 mM) was added. We mixed and withstood for 3 min 1.25 mL of sodium citrate solution (27 mM) and 0.25 mL of AgNO_3_ solution (59 mM). Further, 50 μL of aqueous ascorbic acid solution (102 mM) was injected into the heated water and mixed for 1 min. At the final stage, a mixture of silver source was quickly added to the flask. After 15 min, the resulting solution acquired a bright yellow color, which indicated the formation of silver NPs. The total time of the NPs synthesis was 30 min. At the end, the resulting colloidal solution was centrifuged to remove the by-products of the reaction and unreacted ions. The second part of Ag NPs was synthesized with the SiO_x_ shell structure (Ag/SiO_x_ NPs). For this purpose, we used the modified Stober method [32], with tetraethoxysilane (TEOS) as the source of silicon atoms. Specifically, 20 mL of ethanol and 1.7 μL of TEOS were added to 5 mL of aqueous solution of silver NPs without a shell (core) with active stirring. After 5 min, 1.25 mL of 10% aqueous ammonia solution was added to this mixture. The synthesis time of the silicon oxide shell was 1 h at room temperature.

In order to create hybrid nanostructures, the 50 μL solutions of Ag NPs with and without SiO_x_ were transferred onto the InGaN NWs using a micropipette. Further, the samples were thermally annealed for 20 min at 200 °C and treated in heated acetone for 10 min. This procedure was applied to remove the remnants of the reaction products after the formation of silver NPs.

The morphology of the samples was studied with scanning electron microscopy (SEM) using Supra 25 Carl Zeiss AG. The microstructure and chemical composition of grown NWs were investigated using high-angle annular dark-field scanning transmission electron microscopy (HAADF-STEM, JeolJEM-2100FTEM, Tokyo, Japan) with energy-dispersive X-ray (EDX) spectroscopy techniques (XFlash 6TI30, Bruker, Billerica, MA, USA). The PL measurements were performed at room temperature using a He-Cd laser with a wavelength of 325 nm at 15.5 mW. The laser spot diameter was approximately 100 µm. The PL signal was detected using a MS5204i Sol instruments monochromator and a single-channel Si detector. The LPR of NPs was detected by measuring optical density spectra with a spectrophotometer (Thermo Scientific Multiskan GO, Thermo Fisher Scientific, Waltham, MA, USA). The size of silver NPs was determined using a dynamic light scattering (DLS) Zetasizer Nano ZS setup.

## 3. Results and Discussion

### 3.1. Initial InGaN NWs

**Figure 1:**
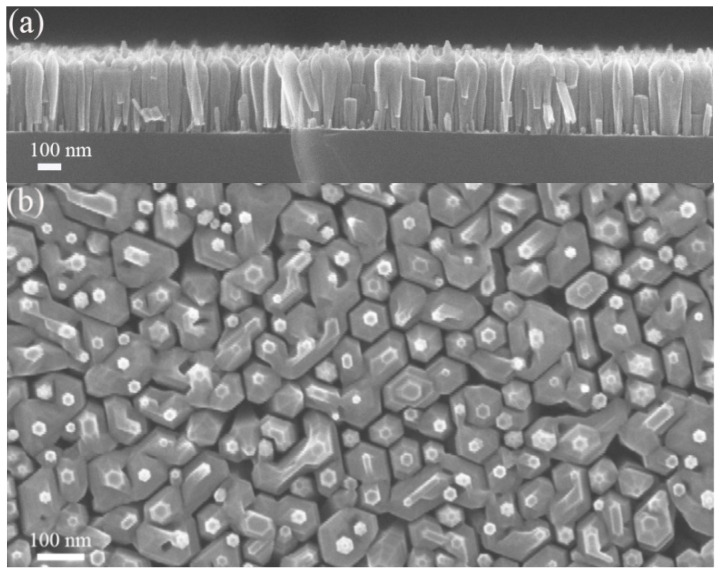
Typical SEM images of InGaN NWs in cross-section (**a**) and plan view (**b**).

Figure 1 shows typical SEM images of the grown InGaN NWs. It is clearly seen that the array contains both separated and partially coalesced NWs with an average height of about 350 nm. The NW diameters are not constant and increase from 30–40 nm at the substrate surface to 80–90 nm in the middle, up to the coalescence of the NW sidewalls. At the top of the structure, the NWs diameter sharply decreases to 20–40 nm, and pyramidal-shaped tips are formed.

**Figure 2:**
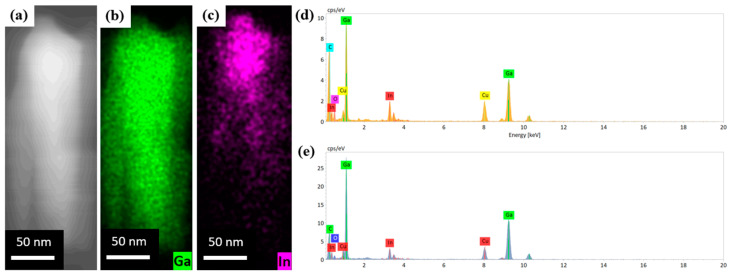
(**a**) Typical HAADF-STEM image of the InGaN NW; (**b**) high-resolution elemental mapping of Ga intensity along the NW; (**c**) high-resolution elemental mapping of In intensity along the NW; (**d**) Typical EDX spectrum of the upper part of NW; (**e**) Typical EDX spectrum of the coalesced part of NW. Green and purple colors correspond to Ga and In, respectively.

To study the structural properties of the grown sample using transmission electron microscopy (TEM), a part of NWs was detached from the silicon substrate and transferred to the Cu grid. We carried out TEM mesaruments for 10 single InGaN nanowires. Figure 2a–c shows typical HAADF-STEM images of the nanowire. The red dots in Figure 1a correspond to the chemical composition obtained through the EDX measurements at these points. The green dots in Figure 2b and the purple dots in Figure 2c show the distribution of Ga and In, respectively. Figure 2d,e show typical EDX spectra measured at the coalesced and the upper part of the NWs. As we can see from Figure 2a–c, chemical composition is unevenly distributed in the NW. Below the coalesced part of the NWs, pure GaN is formed. GaN NWs are formed in the self-induced growth mechanism [33], which is confirmed by the nitrogen-rich conditions and a significant (17% [34]) lattice mismatch between Si (in the (111) direction) and a lattice constant of GaN. At this growth stage, In atoms are not incorporated into NWs, since the InN thermal decomposition and In desorption from the growth surface predominate over the formation of InGaN. In particular, typical temperatures of InN decomposition in vacuum started at 550 °C [35]. Next, it is necessary to take into account the temperature distribution along the NW. It was shown in [36,37,38,39] that the heat transfer from the NWs to the environment is much higher in comparison with the bulk material. Moreover, the formation energy of InGaN on the c-planes of GaN is obviously lower than on the Si(111) surface. In our case, these two factors lead to the incorporation of In into the NWs at the height of ~200 nm, as we can see in Figure 2c. The increase in the NW diameters up to the coalescence occurs due to the local increase in a III/V ratio and a lower diffusion of Ga adatoms in comparison to In [33,40]. Above the coalesced part the diameter of NWs sharply decreases to 20–40 nm and pyramidal-shaped tips are formed. The EDX spectra show that the In content in the tips is about 20–23% against about 10–13% in the coalesced part. As was experimentally and theoretically shown in [41,42], the tip formation on the NW stem denotes the relaxation between materials with a different a lattice constant. We assume that in our case the relaxation process between two areas with different indium contents may result in the formation of tips at the top of the coalesced part of NWs. The C and O lines in the EDX spectra arise from lacey carbon films on the Cu grid (Figure 2d,e).

**Figure 3:**
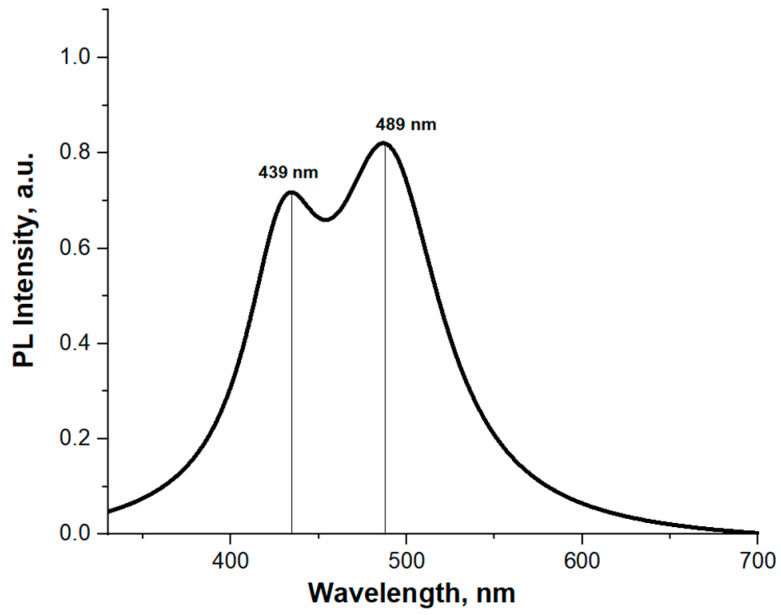
RT PL spectrum of initial InGaN NW array. The short-wavelength PL and long-wavelength PL are attributed to the coalesced part of the NWs and the tips in the upper part of the NWs, respectively.

Figure 3 demonstrates room temperature PL spectrum of the grown sample. The sample exhibits a wide photoluminescence signal from 380 to 570 nm with two maxima corresponding to a short-wavelength peak (439 nm) and a long-wavelength peak (489 nm). The intensities of the maxima differ by 15%.

Let us discuss the peculiarities of the observed PL spectrum. Using the modified Vegard’s law $(E_{g\;InGaN}=xE_{g\;InN}+\left(1-x\right)E_{g\;GaN}-bx\left(1-x\right)$) [43,44,45], one can estimate the dependence of the bandgap energy on the chemical composition of InGaN. According to the TEM results and modified Vegard’s law, the bandgap energy of In_0.1–0.13_Ga_0.9–0.87_N and In_0.2–0.23_Ga_0.8–0.77_N are close to 2.9–3.0 eV (~420 nm) and 2.56–2.67 eV (~475 nm), respectively. Taking into account the Stokes shift for InGaN nanowires (~0.17 eV) [5] and a temperature dependence of PL maxima (~0.07 eV) [46,47] the RT PL of In_0.1–0.13_Ga_0.9–0.87_N and In_0.2–0.23_Ga_0.8–0.77_N should be located near 450 nm and 510 nm. Moreover, the peak position of bandgap energy depends on the internal elastic strains [48,49]. Thus, obtained TEM results fit well into our PL spectra, and we attribute the short-wavelength peak of PL to the coalesced part of the NWs and the long-wavelength peak to the tips in the upper part of the NWs. The bowing parameter was taken equal to 1.43 eV since this value exhibits a good agreement for InGaN nanowires [5,44] and layers [45]. The band gap energy of InN and GaN was 0.7 and 3.4 eV, respectively [43,44,45].

### 3.2. Ag NPs

**Figure 4:**
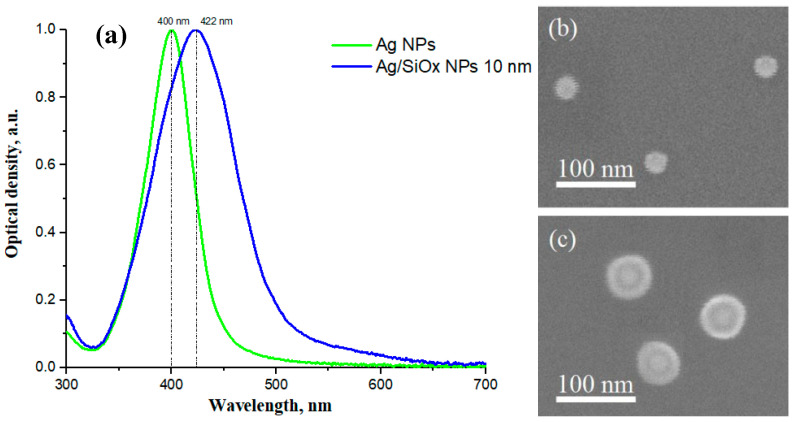
(**a**) The optical density of silver NPs solutions and silver NPs with a silicon oxide shell; typical SEM images of (**b**) Ag NPs and (**c**) Ag NPs with a silicon oxide shell.

Figure 4 shows the optical density spectra of colloidal solutions of Ag NPs and Ag/SiO_x_ NPs. The wavelengths of the spectral maxima correspond to the wavelength of the LPR $\lambda_{LPR}$ in the particles. The $\lambda_{LPR}$ shifts to the red region of the spectrum when a silicon oxide shell is deposited. In the dipole approximation, $\lambda_{LPR}$ of a metal NP depends on the magnitude of the localized charge inside the NP when the plasmon is excited by incident light; the greater the value of this charge, the smaller $\lambda_{LPR}$ is (the greater the energy of the LPR). If the NP is in a dielectric medium, then the charge of the NP is partially compensated due to the polarization effect. With an increase in the dielectric constant of the medium $\varepsilon_{m}$, the effect of polarization increases, which shifts $\lambda_{LPR}$ to the long-wavelength region [50]. In our case, $\varepsilon_{SiO_{x}}$ (for Ag/SiO_x_ NPs) is greater than $\varepsilon_{H_{2}O}$ (for Ag NPs) in the entire visible wavelength range [51,52].

The full width at half maximum (FWHM) of Ag NPs is 53 nm, which indicates the monodispersity of the synthesized NPs [53]. After deposition of the oxide shell, the FWHM is 92 nm. An increase in the FWHM is associated with a change in the permittivity of the NP environment [54], and with the uneven thickness of the oxide shell, as will be shown below. On the other hand, such relative low values of FWHM may indicate a relatively large dephasing time of the LPR in synthesized NPs [55]. This fact predicts the high local-field enhancement factor and a crystalline structure of Ag and Ag/SiOx NPs [55,56].

The optical interaction of InGaN-based nanostructures with metal nanoparticles is achieved when the luminescence wavelength $\lambda_{lum}$ is close to the $\lambda_{LPR}$ [24,57]. In our case, the $\lambda_{LPR}$ of Ag NPs and $\lambda_{LPR}$ of Ag/SiO_x_ are about 400 and 422 nm, respectively (see Figure 4a), which corresponds to the low-energy tails of InGaN PL.

In order to study the NPs using SEM, colloidal solutions were dried on a silicon substrate. Figure 4b,c demonstrates typical SEM images of synthesized NPs with and without the shell. Both types of particles are spherical. The average diameter of silver NPs is about 32 nm. The DLS results show that the colloidal solution with Ag NPs contains only one size fraction of NPs with an average diameter of 30 nm and a polydispersity index of 25%. This range of NP sizes correlates well with the NP diameter of 32 nm obtained using SEM (Figure 4b). The average diameter of Ag/SiO_x_ NPs is about 59 nm (Figure 4c).

### 3.3. Hybrid Nanostructures Based on NWs and NPs

**Figure 5:**
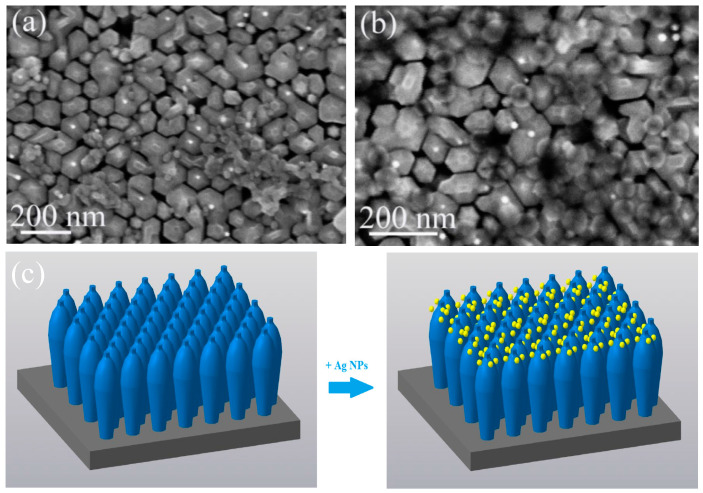
Plan-view SEM images of (**a**) sample 1 and (**b**) sample 2; (**c**) schematical image of the InGaN NW array before and after NPs deposition.

Figure 5 shows typical plane-view SEM images of the InGaN NWs with NPs. The NW arrays with deposited Ag NPs and Ag/SiO_x_ NPs are marked as sample 1 and sample 2, respectively. It is important to note that the average size of NPs is larger than the average distance between NWs (see Figure 1 and Figure 4) at the coalesced part. Therefore, both Ag and Ag/SiO_x_ NPs were not falling down on the substrate. Moreover, NPs are located only on the coalesced part of NWs as we can see in Figure 5a,b. Figure 5c demonstrates schematical images of initial InGaN NWs before and after deposition of NPs.

**Figure 6:**
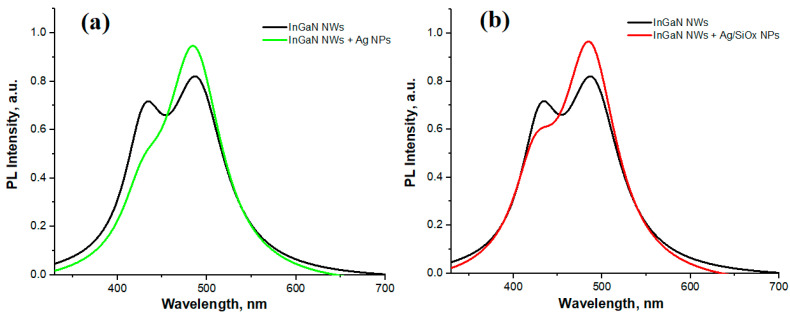
RT PL spectra of sample 1 (**a**) and sample 2 (**b**).

Let us consider the RT PL results of InGaN NWs with Ag and Ag/SiOx NPs. Figure 6 demonstrates the corresponding spectra for sample 1 (a) and sample (b) in comparison with the RT PL of initial sample. It is found that the short-wavelength maxima decreased by 38% for sample 1 (Figure 6a) and by 20% for sample 2 (Figure 6b), whereas the long-wavelength maxima increased by 16 and 19%, respectively. This redistribution of maxima affects the integral intensity in the following way: it decreases by 8–9% for sample 1 and does not change for sample 2.

To explain the observed redistribution of photoluminescence between low and high energy maxima of InGaN NWs, a model was developed.

### 3.4. Model

When the radius of a nanoparticle *r* is much smaller than the wavelength *λ* (*r < < λ*), the light scattering mechanism can be described by the Fröhlich resonance approximation (dipole scattering resonance) [58,59,60,61]. The resonant feature in the spectra of spherical metal nanoparticles will take place near the wavelength at which the Fröhlich condition is satisfied:

(1) $$Re\left\{\varepsilon \left(\omega \right)\right\}=-2Re\left\{\varepsilon_{m}\left(\omega \right)\right\}$$

where ε is the dielectric constant of the metal, and $\varepsilon_{m}$ is the medium. Thus, the equation for Fröhlich resonance for core-shell structure becomes:

(2) (Re{ε2(ω)}+2Re{εm(ω)})(Re{ε1(ω)}+2Re{εm(ω)})=−2(rinrout)3(Re{ε2(ω)}−Re{εm(ω)})(Re{ε1(ω)}−Re{ε2(ω)})

where $\varepsilon_{1}$ is the dielectric constant of the metal, $\varepsilon_{2}$ is the dielectric constant of the shell, $r_{in}$ is the metal core radius and $r_{out}$ is the radius of the entire sphere.

In the first case, the polarizability of a bare metal particle can be written as:

(3) $$\alpha =r^{3}\cdot \frac{\varepsilon -\varepsilon_{m}}{\varepsilon +2\varepsilon_{m}}$$

For a metal particle in a dielectric shell, it will take the form of [62]:

(4) α=rout3·(ε2−εm)(ε1+2ε2)+(rinrout)3(ε1−ε2)((εm+2ε2)(ε2+2εm)(ε1+2εm)+2(rinrout)3(ε2−εm)(ε1−ε2)

**Figure 7:**
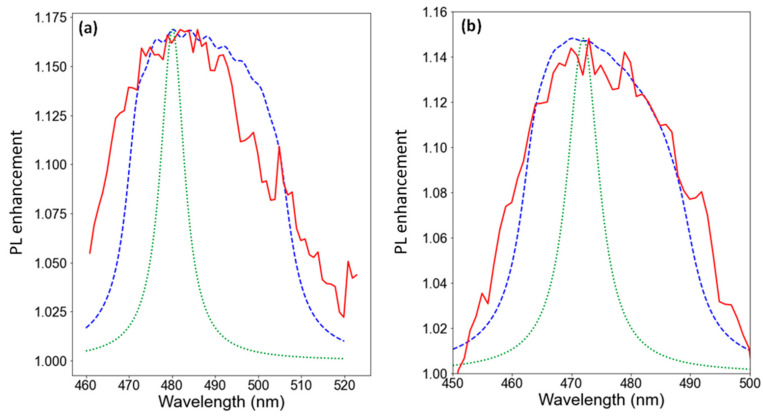
Dependences of the PL enhancement by plasmon nanoparticles on the wavelength. Subfigures (**a**,**b**) correspond to the cases of Ag nanoparticles and core-shell Ag/SiO_x_ nanoparticles. Red lines show the ratios of experimentally measured PL spectra of InGaN nanowires with and without plasmonic nanoparticles. Green dotted lines show theoretically predicted enhancement of PL spectra by the plasmonic nanoparticle surrounded by uniform media with refractive index 2.45. Blue dashed lines demonstrate broadening of calculated PL enhancement spectra in the case of normal spread of effective refractive index of the surrounding media in the range (2.45 ± 0.1).

Calculated enhancements of PL intensity via the Fröhlich resonance for the single plasmonic nanoparticle surrounded by uniform media correlate with polarizabilities of plasmonic nanoparticle defined by Equations (3) and (4). Figure 7a,b shows the dependences of the PL enhancement by plasmon nanoparticles on the wavelength. The green dotted lines correspond to the theoretically predicted enhancement of PL spectra by the plasmonic nanoparticle surrounded by uniform media with refractive index 2.45. It should be noted that the core-shell structure (Figure 7b) shows a blue shift in respect to silver nanoparticle. To calculate the refractive index, we have used the modified Vegard’s law in accordance with the results in [45,62,63]. The refractive index for InN and GaN was 3.05 and 2.57, respectively. The bowing parameter was equal to 1.43 eV. The refractive index of 2.45 was calculated for InGaN with an In content of 16%.

As we can see from Figure 7, the experimentally observed enhancement of PL intensity (red solid lines) expressed as ratio of PL spectra with and without plasmonic nanoparticles has much larger spectral width than the Fröhlich resonance. In the system under consideration, the InGaN structure was not homogeneous. There is variation of chemical composition within a nanowire, and the particles can be in different positions in respect to the coalesced part of NWs and tips, which leads to a broadening of the spectrum, as can be seen in Figure 7. As a result, surrounding media cannot be considered as uniform, and Equations (1)–(4) should deal with effective refractive index distributed within certain interval. Taking into consideration the different In content between the coalesced part of the NWs and the tips, we estimate that the spread of refractive index equals to 0.1. The calculated PL enhancement with effective refractive index characterized by mean value 2.45 and spread 0.1 is shown by blue dashed line (see Figure 7), and there is good correspondence between the experimental dependence and the results of the modeling.

The observed luminescence quenching in the short-wavelength region can be explained by the diffusion of charge carriers (electrons and holes) between areas with different indium contents. The literature describes the effect of nanosized inclusions on the diffusion of charge carriers and recombination rates [64,65]. In regions that provide a long-wavelength peak of NW photoluminescence due to the chemical composition, a higher recombination rate takes place due to the influence of the proximity of the resonant feature of the scattering spectrum of nanoparticles. In the long-wavelength region, the concentration of charge carriers is depleted faster, which leads to their diffusion from regions of alternative chemical composition. Thus, in the short-wavelength region, the stock of charge carriers is reduced compared to the situation when there are no particles on the NW, which leads to the experimentally demonstrated quenching of the luminescence peak in the shortwave region of the spectrum. Moreover, this difference in PL quenching can be explained by the absence of a silicon oxide shell in sample 1. This result is consistent with the data in [66], in which the nonradiative quenching process prevails in the absence of a gap between the metal NP and the emitter.

**Figure 8:**
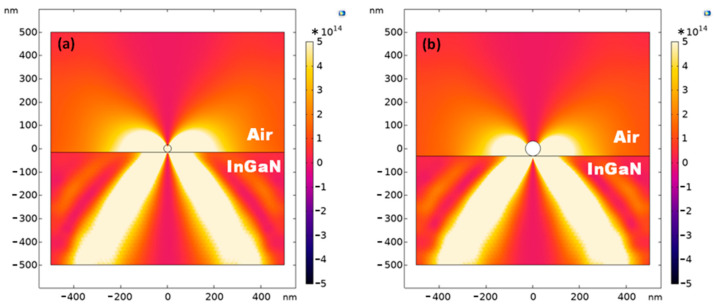
The distribution of the Poynting vector modulus in the process of light scattering on a nanoparticle at the interface between air and InGaN nanowire: (**a**) silver nanoparticle, λ = 485 nm. (**b**) core-shell nanoparticle, λ = 473 nm. Figure shows the cut plane of a nanoparticle located on the surface of a nanowire (the interface between air and InGaN). The x coordinate is parallel to the nanowire, and the y coordinate is perpendicular. Both are expressed in nm.

Light scattering on a particle with and without an additional coating occurs in accordance with similar physical principles (see Equations (3) and (4)); however, the presence of a dielectric coating shifts the resonance frequency. Figure 8 shows the distributions of the Poynting vector at resonance frequencies in the cut plane of a nanoparticle located on the surface of a nanowire (the interface between air and InGaN).

## 4. Conclusions

To conclude, we have synthesized InGaN NWs on the Si substrate via plasma-assisted MBE. The obtained sample has short-wavelength and long-wavelength RT PL peaks in the blue region of the visible spectrum. Based on the STEM and SEM studies, we associated these two peaks with a different In content in the coalesced area (10–13%) and the tips (20–23%) of InGaN NWs. After the deposition of colloidal Ag and Ag/SiO_x_ NPs on the NW surface, we have observed a redistribution of PL in both cases. Specifically, this effect is associated with the quenching of the short-wavelength peak and enhancement of the long-wavelength peak. This enhancement was explained by the Fröhlich resonance for silver NPs surrounded by a medium with a refractive index of 2.45 and a spread of 0.1. We have attributed the quenching of the short-wavelength peak to the diffusion of charge carriers from the areas with 10–13% In content to the areas with 20–23% In content. Thus, the decoration of silver NPs on the surface of InGaN NWs with an inhomogeneous distribution of In makes control of the luminescent properties of the latter possible. The proposed approach to controlling the luminescence properties of InGaN NWs with a nonuniform distribution of In can be useful for the fabrication of light-emitting devices based on NWs.

## Data Availability

Not applicable.
